# Molecular Insights into the Accelerated Sprouting of and Apical Dominance Release in Potato Tubers Subjected to Post-Harvest Heat Stress

**DOI:** 10.3390/ijms25031699

**Published:** 2024-01-30

**Authors:** Tengfei Liu, Qiaoyu Wu, Shuai Zhou, Junhui Xia, Wang Yin, Lujun Deng, Botao Song, Tianjiu He

**Affiliations:** 1 College of Food Science and Engineering, Shandong Agricultural University, Taian 271018, China; hzauzsmj@gmail.com; 2 Institute of Biotechnology, Guizhou Academy of Agricultural Sciences, Guizhou Key Laboratory of Agricultural Biotechnology, Key Laboratory of Crop Genetic Resources and Germplasm Innovation in Karst Mountainous Areas, Ministry of Agriculture and Rural Affairs, Guiyang 550025, China; wuqiaoyu1@163.com (Q.W.); zhous202310@163.com (S.Z.); yinwang_2023@foxmail.com (W.Y.); denglujun@163.com (L.D.); 3 National Key Laboratory for Germplasm Innovation & Utilization of Horticultural Crops, Key Laboratory of Potato Biology and Biotechnology (HZAU), Ministry of Agriculture and Rural Affairs, Potato Engineering and Technology Research Center of Hubei Province, College of Horticulture and Forestry Science, Huazhong Agricultural University, Wuhan 430070, China; xiajh@mail.hzau.edu.cn (J.X.); songbotao@mail.hzau.edu.cn (B.S.)

**Keywords:** *Solanum tuberosum*, potato sprouting, abiotic stress, transcriptome, transcription factor, apical bud meristems

## Abstract

Climate change-induced heat stress (HS) increasingly threatens potato (*Solanum tuberosum* L.) production by impacting tuberization and causing the premature sprouting of tubers grown during the hot season. However, the effects of post-harvest HS on tuber sprouting have yet to be explored. This study aims to investigate the effects of post-harvest HS on tuber sprouting and to explore the underlying transcriptomic changes in apical bud meristems. The results show that post-harvest HS facilitates potato tuber sprouting and negates apical dominance. A meticulous transcriptomic profiling of apical bud meristems unearthed a spectrum of differentially expressed genes (DEGs) activated in response to HS. During the heightened sprouting activity that occurred at 15–18 days of HS, the pathways associated with starch metabolism, photomorphogenesis, and circadian rhythm were predominantly suppressed, while those governing chromosome organization, steroid biosynthesis, and transcription factors were markedly enhanced. The critical DEGs encompassed the enzymes pivotal for starch metabolism, the genes central to gibberellin and brassinosteroid biosynthesis, and influential developmental transcription factors, such as SHORT VEGETATIVE PHASE, ASYMMETRIC LEAVES 1, SHOOT MERISTEMLESS, and MONOPTEROS. These findings suggest that HS orchestrates tuber sprouting through nuanced alterations in gene expression within the meristematic tissues, specifically influencing chromatin organization, hormonal biosynthesis pathways, and the transcription factors presiding over meristem fate determination. The present study provides novel insights into the intricate molecular mechanisms whereby post-harvest HS influences tuber sprouting. The findings have important implications for developing strategies to mitigate HS-induced tuber sprouting in the context of climate change.

## 1. Introduction

The potato (*Solanum tuberosum* L.) is the most important non-cereal food crop and plays a crucial role globally in alleviating the increasing food demand driven by rapid population growth. To meet the year-round demand for fresh consumption, tubers require prolonged post-harvest storage [1]. Sprouting is one of the primary contributors to quality degradation during tuber storage, causing the redistribution and metabolism of stored substances and shrinkage due to water loss [1]. Therefore, comprehending the physiological and molecular mechanisms underlying tuber sprouting and developing technological methods to regulate post-harvest tuber sprouting are vital for ensuring the success of long-term tuber storage.

Potato tubers develop from underground stolon, typically emanating from the basal stem nodes, and represent a diageotropic shoot or stem characterized by significantly extended internodes [2]. Serving as a modified form of an underground stolon, the potato tuber typically acts as a model for investigating the physiological and biochemical processes associated with dormancy release, sprouting initiation, and aging. The activation status of the apical and axillary buds of the tuber significantly influences vital agronomic traits, including tuberization, dormancy, and sprout number [3]. The status of tuber buds depends on the meristematic activity; for instance, when the meristematic activity completely ceases, the buds enter dormancy, while the onset of tuber sprouting requires the reactivation of meristematic activity [4]. On the cellular scale, dormancy primarily manifests as a halt in the G_1_/G_0_ phase of the meristematic cells [5]. Dormancy termination is linked with the reactivation of the meristem, characterized by a re-entry into the G1 phase, and subsequently facilitating DNA replication in the S phase, which paves the way for bud emergence. Deoxyuridine triphosphatase (dUTPase) plays a central role during the DNA replication process; its expression signifies the transition from dormancy to sprouting in potato tuber buds, serving as a marker for meristem reactivation in tuber buds [5,6]. Moreover, cell replication markers, such as histone *H4* and D-type cyclin *CYCD3*s, are also induced during the transition from dormancy to sprouting [7]. The studies above demonstrate that the transcriptional regulation of cell division-related genes and the reactivation of meristematic tissues are intricately associated with the release from dormancy.

The frequent occurrence of heat stress (HS), driven by climate change and global warming, poses a significant threat to potato production. HS affects tuberization and results in heat sprouting, where immature tubers frequently sprout during growth when plants are cultivated during hot seasons [8]. If potato tubers sprout either prior to harvest or shortly after that, their quality and utility diminish significantly. Thus, gaining insight into the influence of HS on potato tuber sprouting and its related gene regulatory mechanisms becomes imperative. Significant progress has been made in deciphering the impact of HS on potato sprouting. Observations have indicated that when potatoes in the field are exposed to HS, their tubers might experience a reduced or absent dormancy phase [9]. In comparison, unstressed tubers typically sprout only after several months in post-harvest storage [10]. HS can trigger the emergence of heat sprouts, curtailing the duration of post-harvest dormancy [11]. Notably, tubers that underwent sprouting due to heat have been found to enter a period of dormancy once harvested [11]. Previous studies have predominantly focused on the effects of pre-harvest HS on sprouting, while the impacts of post-harvest HS on tubers remain largely unexplored.

The application of high-throughput mRNA sequencing (RNA-Seq) has facilitated the gene expression analysis of cultivar Russet Burbank tubers in post-harvest dormant and normally sprouted stages [7], alongside that of other cultivars [6,12,13,14]. A substantial number of differentially expressed genes (DEGs) have been identified between the dormant non-sprouted and post-dormancy sprouting phases in these studies, revealing that the post-harvest dormancy to post-dormancy sprouting transition encompasses various physiological processes and the differential modulation of numerous genes. However, compared to the tubers that sprout normally post-harvest, it remains unclear whether mature tubers subjected to post-harvest HS display comparable responses regarding DEGs, biological processes, and metabolic pathways.

Building upon the aforementioned findings, it is hypothesized that HS potentially modifies the sprouting behavior of post-harvest potato tubers. To test this hypothesis, the core objectives of this investigation include (1) exploring the impact of post-harvest HS on potato tuber sprouting patterns; (2) conducting a detailed transcriptomic analysis to chart the shifts in sprouting dynamics under HS; (3) highlighting the pivotal pathways and DEGs responsive to HS-triggered sprouting changes; and (4) gaining insights into the underlying molecular mechanisms governing HS-induced alterations in sprouting behavior. This research fills a gap in the current understanding of post-harvest heat stress (PHHS) effects on potato tubers from a molecular and physiological perspective.

## 2. Results

### 2.1. Post-Harvest HS Promotes Tuber Sprouting and the Release of Apical Dominance

**Figure 1:**
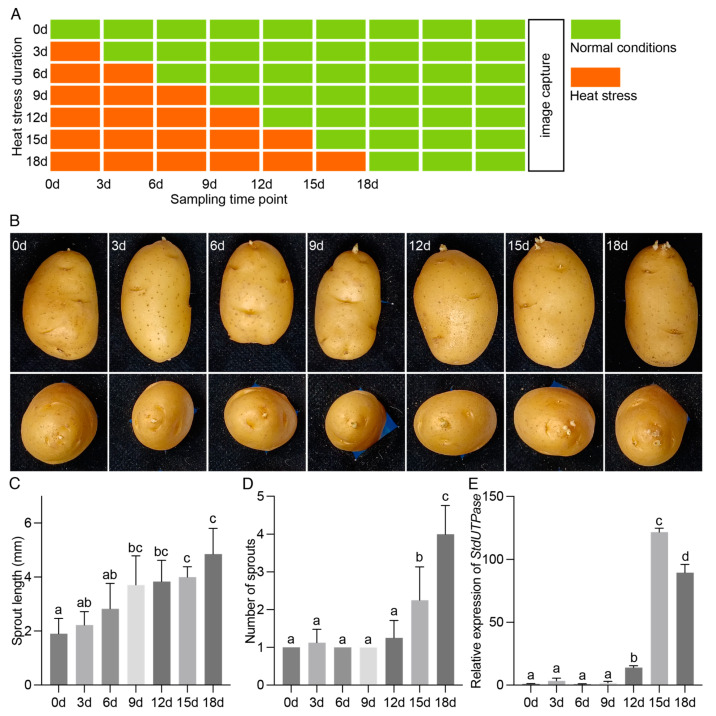
Effects of post-harvest HS on potato sprouting. (**A**) Schematic diagram of the post-harvest HS treatment on potatoes. (**B**) Phenotypes of potato sprouting at different times after the HS treatment, with the treatment details as shown in A, and photographs taken 9 days after the end of the treatment. (**C**) Statistics for sprout length after different durations of HS treatment. (**D**) Statistics for the number of sprouted buds (length > 1 mm) after various durations of HS treatment. (**E**) Expression profiles of *StdUTPase* in tuber apical bud meristem after various durations of HS treatment. Data are presented as means ± SD (n = 3). Different lowercase letters indicate significant differences (*p* < 0.05; analyzed using one-way ANOVA followed by Tukey’s multiple comparisons test).

To explore the impact of post-harvest HS on tuber sprouting, freshly harvested “Ac142” tubers were stored at 20 °C for two weeks to facilitate the healing of injuries incurred during harvesting. Subsequently, these tubers were subjected to varying durations of HS treatment, with temperatures elevated to 30 °C for a maximum of 18 days. The comprehensive treatment plan is depicted in Figure 1A, which illustrates the different durations which the tubers were exposed to HS before being returned to normal conditions; they were then photographed for evaluation 27 days after the onset of treatment. HS significantly accelerated tuber sprouting, as indicated by the marked increase in sprout length 9 days after the end of the treatment, especially following heat exposure of 9 days or longer, compared to the control (Figure 1B,C). Moreover, concurrent sprouting of multiple apical buds was observed after 15 and 18 days of HS treatment, suggesting the release of apical dominance (Figure 1B). Statistical results for the number of sprouts per tuber show that a HS treatment of 12 days or fewer did not affect the number of sprouts (Figure 1D). However, HS treatment that reached 15 days significantly increased the number of sprouts per tuber and, after 18 days of treatment, the number of sprouts per tuber further increased compared to that at 15 days (Figure 1D). The expression profiles of *StdUTPase*, a marker for meristem reactivation in tuber buds [5,6], began to rise on day 12 and showed substantial increases on days 15 and 18 (Figure 1E), indicating meristem reactivation even without visible sprouting. These results reveal that post-harvest HS promotes tuber sprouting and apical dominance release, and this promoting effect seems to require the accumulation of a certain amount of heat exposure.

### 2.2. Dynamic Transcriptome Profiling of Tuber Apical Bud Meristematic Tissue during HS Treatment

**Figure 2:**
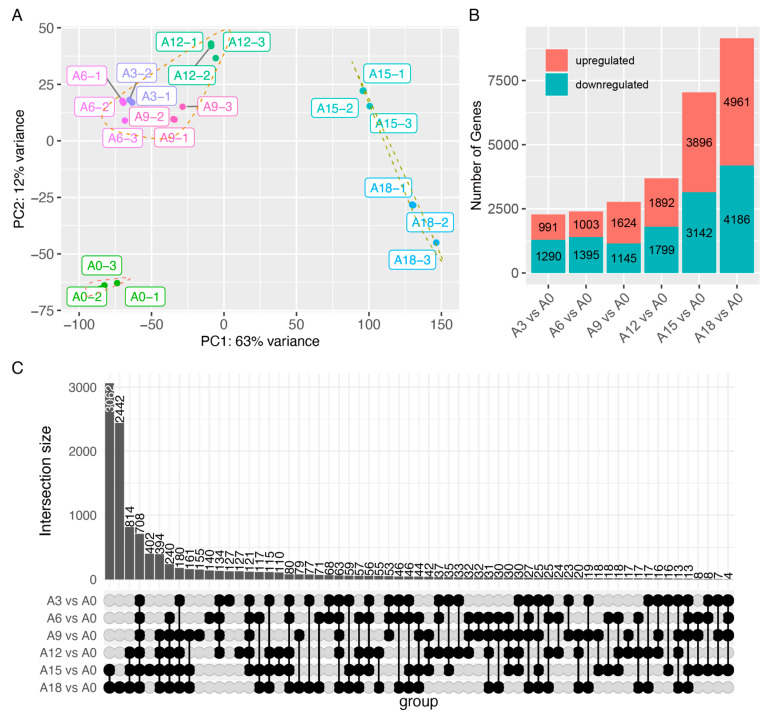
Transcriptomic analysis of tuber apical bud meristem following HS treatment. (**A**) Principal component analysis (PCA) of transcriptomic sequencing samples. (**B**) Comparative analysis of upregulated and downregulated genes between HS treatments and control. (**C**) Upset illustrating DEGs during various HS treatment durations. The upper column displays the intersecting number of DEGs, while the left column graph delineates the number of DEGs in each dataset. Individual black dots represent the DEGs exclusively found in a single dataset. DEGs observed in at least two datasets are denoted by interconnected black dots, with the connections illustrating the intersections, and the black dots on the line indicating the pertinent datasets. The gray dots represent DEGs that are not included in the datasets.

To understand the reasons behind the impact of post-harvest HS on tuber sprouting, the apical bud meristematic tissue of the tubers was sampled at three-day intervals during the HS treatment, starting before the treatment, for RNA extraction and RNA-seq-based transcriptome profiling. The PCA (principal component analysis) results showed that replicate samples from the same group clustered together, except for A3-3, which was excluded from the subsequent analyses. The clustering of the PCA results revealed that the samples could be divided into three clusters: HS treatment for 0 days, HS treatment for 3–12 days, and HS treatment for 15 and 18 days (Figure 2A).

As the duration of the HS treatment extended, the number of DEGs also increased (Figure 2B). After 3 days of treatment, there were only 991 upregulated genes and 1290 downregulated genes, but after 18 days of treatment, there were 4961 upregulated genes and 4186 downregulated genes (Figure 2B). The Upset analysis indicated that the highest number of commonly different DEGs were noted explicitly at 15 and 18 days, totaling 3062 (Figure 2C). Following this, there were 2442 DEGs exclusively expressed at the 18-day mark. Subsequently, 814 DEGs were commonly different for the 12-, 15-, and 18-day periods (Figure 2C). There were 708 genes that exhibited a differential expression across all the examined time frames (Figure 2C). Numerous genes showed specific differential expressions after HS treatment for 15 and 18 days, potentially linked to alterations in the apical bud meristematic tissue state, as both treatments resulted in a loss of apical dominance.

**Figure 3:**
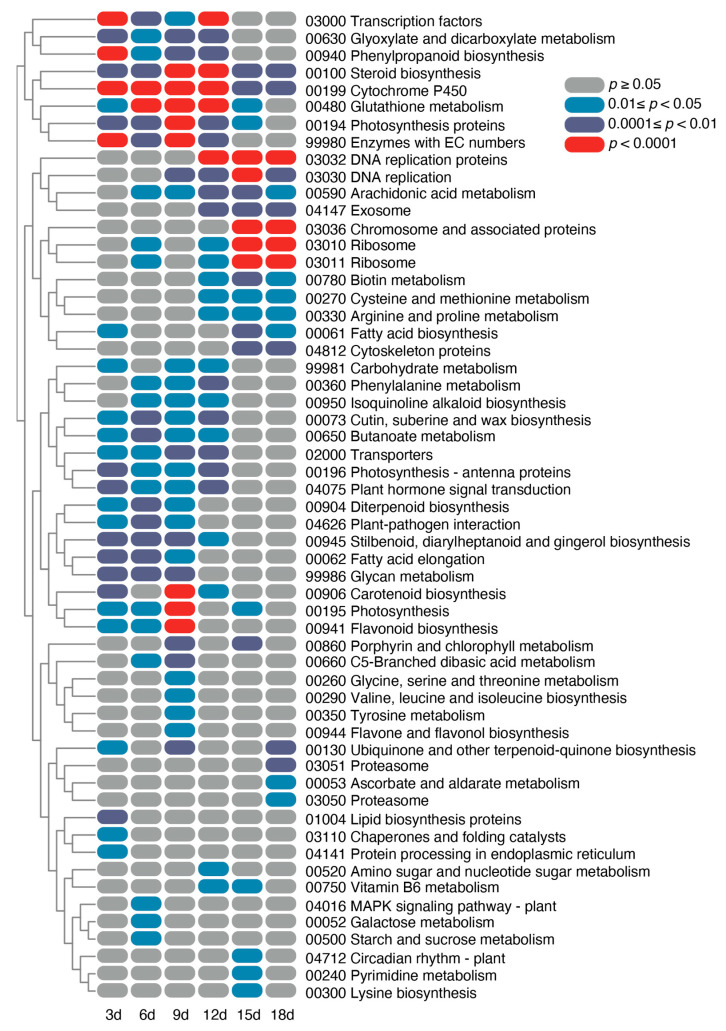
Heatmap illustrating enriched KEGG pathways of DEGs during various HS treatment durations.

A functional enrichment analysis was conducted on these lists of DEGs, utilizing the Kyoto Encyclopedia of Genes and Genomes (KEGG) pathways to gain a deeper insight into the functions of the identified DEGs. As shown in Figure 3, the enrichment of pathways by the DEGs varied across the different HS treatment durations, with most of the KEGG pathways showing enrichment at multiple time points. Generally aligning with the PCA results, the HS treatments spanning 3–12 days and those of 15 and 18 days converged on different common pathways (Figure 3). Specifically, the pathways such as “steroid biosynthesis” and “Cytochrome P450” were enriched for all heat treatment durations, and 10 pathways, including “transcription factors”, “transporters”, and “plant hormone signal transduction”, were particularly enriched during the HS treatments spanning 3–12 days (Figure 3). Notably, the pathways involving “chromosome and associated proteins” and “cytoskeleton proteins” were distinctively enriched at the 15- and 18-day HS treatment marks (Figure 3). Considering the loss of apical dominance in the potato apical bud meristematic tissue at these stages, these two pathways might be related to this phenomenon.

### 2.3. Identification of DEGs with a Similar Temporal Expression Pattern

**Figure 4:**
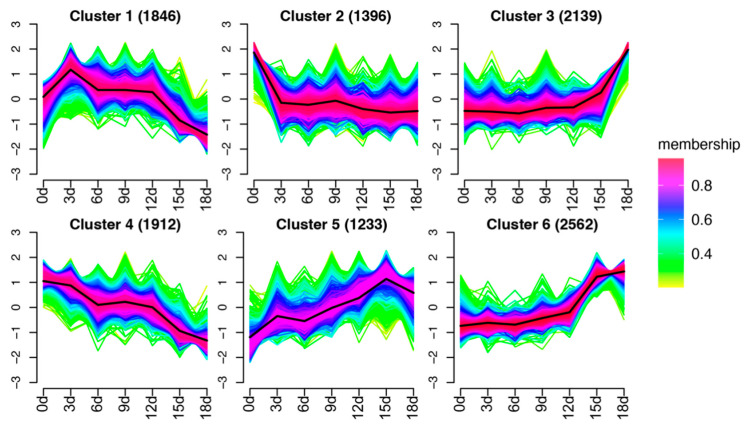
Fuzzy c-means clustering delineates predominant gene expression trends across the six HS treatment time points. The average TPM values of the DEGs are used. In this context, a particular gene’s membership score (MS) within a cluster is depicted through a color spectrum, where red (corresponding to MS  =  1) signifies a high degree of association. The thick black line represents the group center.

A fuzzy c-means (FCM) clustering was employed to delineate the overall expression patterns of the 11,088 DEGs previously mentioned (Table S1). In the FCM clustering approach, every profile is allocated a degree of membership for a group of clusters. A methodical investigation of the various combinations of cluster sizes and fuzzification parameters was conducted, and identified the optimal partitioning when c = 6 and m = 1.75. This partitioning resulted in six clusters. Cluster 1 contained 1846 genes, showing an upregulated expression pattern after 3 days of HS treatment, followed by a downregulated expression (Figure 4). Cluster 2 included 1396 DEGs that were downregulated immediately after the HS treatment, but stabilized throughout the HS process (Figure 4). Cluster 3, comprising 2139 DEGs, initially remained relatively stable but showed a dramatic upregulation after 18 days of heat treatment (Figure 4). Cluster 4, consisting of 1912 DEGs, showed a downregulated expression as the heat treatment progressed (Figure 4). Cluster 5, with 1233 DEGs, exhibited an upregulated expression in the early stages of the HS treatment, but was slightly downregulated after 18 days of HS treatment (Figure 4). Lastly, Cluster 6, which contained 2562 DEGs, began to slowly upregulate after 6 days of HS treatment, followed by a sharp upregulation at 15 days, and a further increase in its expression levels at 18 days (Figure 4). The cluster analysis results indicate that the DEGs exhibited various dynamics during the HS treatment.

### 2.4. Identification of DEGs with a Similar Temporal Expression Pattern

**Figure 5:**
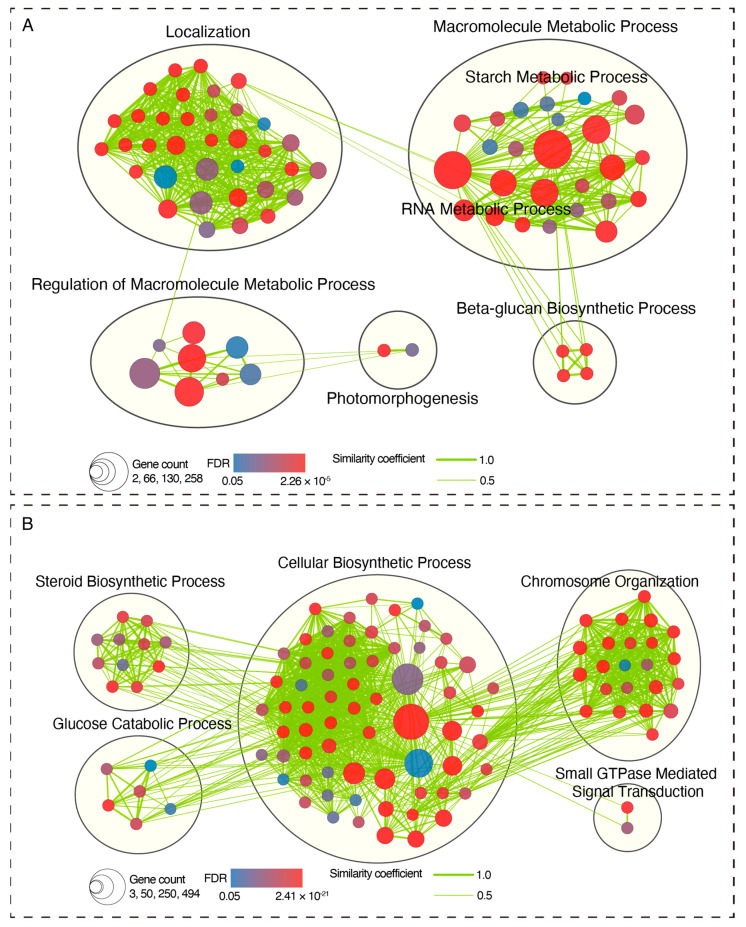
GO enrichment analysis of DEGs in Clusters 4 (**A**) and 6 (**B**). Only biological process categories are shown for this analysis. Nodes denote significantly enriched gene sets, where the fill color indicates the FDR value, the size corresponds to the gene count within the set, and the edges represent the overlapping similarity coefficient.

Attention was particularly given to Clusters 4 and 6, as these clusters exhibited notable specific downregulation and upregulation expression patterns at the critical 15- and 18-day junctures during the HS treatment. Consequently, KEGG and GO enrichment analyses of the DEGs within these two clusters were performed. For Clusters 4 and 6, the enrichment analysis identified 77 and 108 significant biological process (BP) gene sets (Figure 5), respectively. These gene sets coalesced into five distinct pathway clusters in each (Figure 5). For the DEGs in Cluster 4, the predominantly enriched pathways were “Localization” and “Macromolecule Metabolic Process” (Figure 5A). Conversely, the DEGs in Cluster 6 were primarily associated with the pathways including the “Cellular Biosynthetic Process” and “Chromosome Organization” (Figure 5B). The KEGG enrichment analysis revealed that the DEGs in Clusters 4 and 6 notably enriched the development-related signaling pathways, such as the “Circadian Rhythm-Plant” and “Chromosome and Associated Proteins” (Figure S1). These findings suggest that a HS treatment might orchestrate the modulation of apical dominance release by altering the expression of a substantial set of genes in the meristematic tissue of the tuber apical bud.

### 2.5. Identification of the Candidate DEGs Potentially Involved in HS Treatment-Induced Sprouting Variations

**Figure 6:**
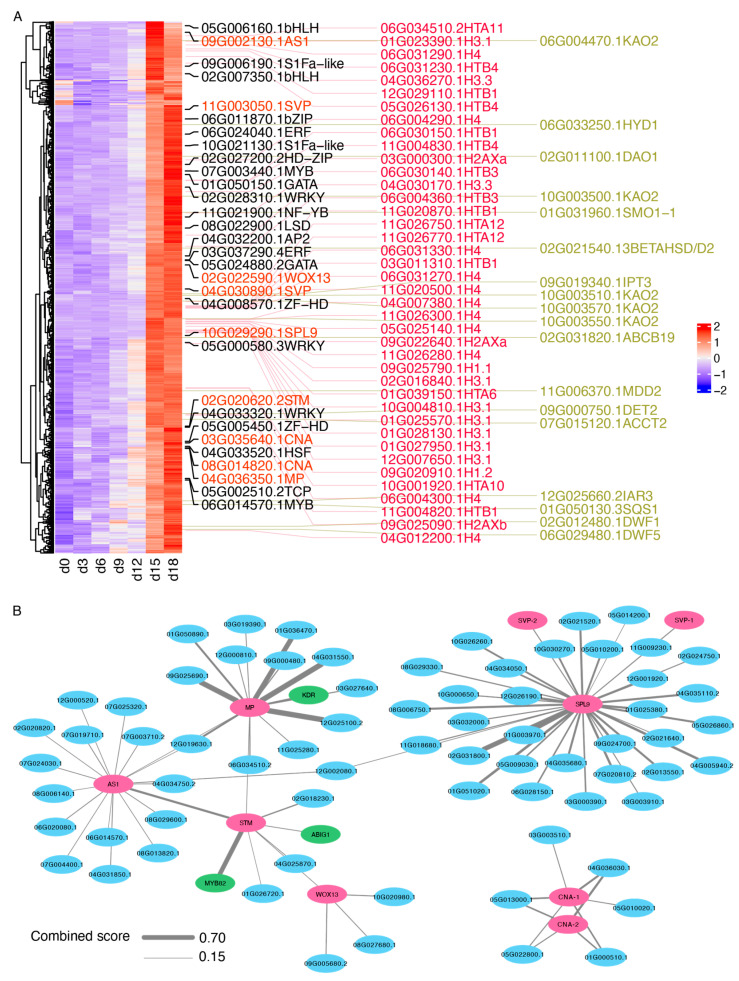
Identifying potential core regulatory genes in HS-induced sprouting variations. (**A**) Heatmap illustration of DEGs in Cluster 6. DEGs encoding TFs (annotated on the left; red indicates potential development-related TFs), histones (annotated in the middle in red), and enzymes (annotated on the right in gold) implicated in the steroid biosynthetic process are highlighted. (**B**) String protein–protein interactions (PPI) network analysis of potential core regulatory TFs within cluster 6. The potential development-related TFs are marked in red, the TFs newly identified through PPI are marked in green, and other non-transcription factor members are marked in blue. The thickness of the nodes represents the value of the combined score. The network was generated using the STRING website (https://string-db.org, accessed on 22 January 2024).

Our efforts were concentrated on identifying the crucial genes potentially responsible for the release of apical dominance induced by the HS treatment, emphasizing the transcription factors and DEGs involved in the critical pathways found in Clusters 4 and 6. Within Cluster 4, the pathways that included the “Starch Metabolic Process”, “Photomorphogenesis”, and “Circadian Rhythm-Plant” were the primary focus (Figure 5A and Figure S1). Seven genes encoding essential enzymes were highlighted within the “Starch Metabolic Process”, including the entities associated with starch synthesis, such as *ADP-glucose pyrophosphorylase* (*AGPase*) and *granule-bound starch synthase* (*GBSS*), and those linked to degradation processes, like *α-Glucan, water dikinase* (*GWD*), and *phosphoglucan, water dikinase* (*PWD*). For “Photomorphogenesis” and “Circadian Rhythm-Plant”, the significant genes comprised *FLOWERING LOCUS C* (*FLC*), *PSEUDO-RESPONSE REGULATOR* (*PRR*), *CIRCADIAN CLOCK ASSOCIATED 1* (*CCA1*), *SWINGER* (*SWN*), and *TIMING OF CAB EXPRESSION 1* (*TOC1*) (Figure S2). Notably, these genes experienced a downtrend on the HS treatment’s 15th and 18th day (Figure S2). For Cluster 6, the focus was also on the pathways of “Chromosome Organization” and “Steroid Biosynthetic Process”, in addition to the transcription factors. For the “Chromosome Organization” pathway, we identified 40 DEGs that encode histones and their variants (Figure 6A). For the “Steroid Biosynthetic Process”, five genes were annotated as Ent-kaurenoic acid oxidase 2 (KAO2), a pivotal enzyme in the gibberellins (GAs) biosynthesis pathway, along with three DEGs that encode crucial enzymes in the BR synthesis pathway—deetiolated2 (det2), dwarf1 (DWF1), and DWF5—and were specifically upregulated on the 15th and 18th day of the HS treatment (Figure 6A). Additionally, potential development-related TFs were identified, including *SHORT VEGETATIVE PHASE* (*SVP*), *WUSCHEL RELATED HOMEOBOX 13* (*WOX13*), *ASYMMETRIC LEAVES 1* (*AS1*), *SQUAMOSA PROMOTER BINDING PROTEIN-LIKE 9* (*SPL9*), *SHOOT MERISTEMLESS* (*STM*), *MONOPTEROS* (*MP*), and *CORONA* (*CNA*), among others (Figure 6A). The expression profiles of the nine TFs were further validated using qRT-PCR, which showed good concordance with the RNA-seq results (Figure S3), further confirming the reliability of the RNA-seq findings. Simultaneously, the STRING database was utilized to analyze the interaction networks of these potential regulatory transcription factors, identifying new TFs possibly involved in the following potential key regulatory factors’ interactions in Cluster 6: *ABA INSENSITIVE GROWTH 1* (*ABIG1*), *KIDARI* (*KDR*), and *MYB82* (Figure 6B).

## 3. Discussion

### 3.1. The Impact of HS Treatment on Post-Harvest Potato Tuber Sprouting

In the context of escalating global climate change, the impact that HS exerts upon crops is intensifying. The potato, recognized for its sensitivity to HS and as a major staple food worldwide, finds itself at the crossroads of mounting challenges concerning diminished growth and compromised yield quality. Additionally, understanding the impact of HS on potato sprouting is beneficial for addressing the future post-harvest quality maintenance of potatoes. Clarifying the mechanisms through which HS promotes sprouting can aid in developing strategies to suppress tuber sprouting under high-temperature conditions. Therefore, it becomes particularly crucial to unravel the complex mechanisms underlying the effects of HS on potato tuber sprouting. In recent years, there have been significant strides in understanding HS’s effects on potatoes’ sprouting. It has been noted that the HS experienced by potato crops in the field can potentially reduce or even eliminate the dormancy period of the tubers [9]; in contrast, tubers that have not been subjected to heat stress usually only begin to sprout after several months in post-harvest storage [10]. It has been found that HS alone can induce the development of heat sprouts, shortening the period of post-harvest dormancy [11]. Interestingly, the tubers that sprouted due to heat later entered a dormant state post-harvest [11]. However, the studies mentioned above were targeted at pre-harvest potato plants. To the best of our knowledge, the present research is the first to evaluate the impact of HS on post-harvest tuber sprouting, substantiating the claim that a post-harvest HS treatment can accelerate sprouting and facilitate the release of apical dominance. This approach posits that HS treatments could serve as a natural substitute for chemical sprout suppressants, especially beneficial in organic farming, by efficiently breaking the post-harvest dormancy of seed tubers. However, the ideal conditions for this technique still necessitate further refinement. Additionally, the continued development of the sprouts induced by HS and the status of the plants developed from these sprouts appear to be normal (Figure S4). However, to substantiate this conclusion will require further support from detailed statistical data and results from multi-year field cultivations.

### 3.2. Transcriptome Unveils Potential Mechanisms of HS Affecting Potato Sprouting

It has been established that post-harvest HS accelerates tuber sprouting and facilitated apical dominance release, with the degree of this promotional effect appearing to depend on the duration of the HS exposure. Therefore, specific genes and biological processes are anticipated to respond to HS treatments of different periods. The biological pathways enriched during the initial phase of the HS treatment (3–12 days) exhibited substantial similarities, and a parallel trend was observed in the later stage, with a marked resemblance in the enriched pathways discerned on both the 15th and 18th day of the HS treatment.

A cluster analysis of these DEGs identified two distinct gene sets that were downregulated and upregulated on the 15th and 18th day of the HS treatment. The downregulated gene set was enriched with starch metabolism, photomorphogenesis, and Circadian Rhythm–Plant pathways. Previous research has substantiated the pivotal role of starch metabolism in regulating potato sprouting [15], illustrating that the concurrent suppression of isoamylases *ISA1*, *ISA2*, and *ISA3* can accelerate sprouting [16], whereas inhibiting α-amylase *StAmy23* tends to delay sprouting [17]. Photomorphogenesis and Circadian Rhythm–Plant pathways play a crucial role in regulating plant development. For instance, in Arabidopsis, the FLC can concurrently modulate the flowering time and seed germination [18,19]; similarly, plant circadian clock genes, like LATE ELONGATED HYPOCOTYL (LHY), CCA1, and GIGANTEA (GI), occupy a central position in governing the seed dormancy and germination processes, and mutations in these genes can hinder seed germination in cold temperatures [20].

The upregulated gene set manifested an enrichment of the pathways including chromosome organization and steroid biosynthetic process, wherein a significant number of histones and their variants implicated in chromosome organization play pivotal roles in plant responses to HS and seed germination [21,22]. The steroid biosynthetic process encompasses vital plant hormone metabolic pathways, including gibberellin (GA) and brassinosteroid (BR) synthesis. Five DEGs enriched in the steroid biosynthetic process were annotated as encoding KAO2, a type of cytochrome P450 monooxygenases belonging to the CYP88A subfamily, which facilitates the transformation of ent-kaurenoic acid (KA) into gibberellin GA12, the progenitor of all GAs [23]. Furthermore, GAs can break tuber dormancy and promote sprouting [4]. In addition, *Det2*, *DWF1*, and *DWF5,* which encode Steroid 5-alpha-reductase, Delta(24)-sterol reductase, and 7-dehydrocholesterol reductase, respectively—essential enzymes in the biosynthesis of BRs [24,25,26]—also exhibited high expression levels on the 15th and 18th day of the HS treatment. BRs treatment has also been found to promote potato sprouting and tomato AD release [27,28]. Therefore, it is rational to hypothesize that HS treatment facilitates tuber sprouting and AD release by inducing the expression of key enzyme genes in the synthesis processes of GAs and BRs, subsequently promoting the accumulation of these substances.

Transcription factors (TFs) are considered global regulators of plant development and responses to the environment [29]. The current study identified 31 TFs that exhibited high expression levels on the 15th and 18th day of the HS treatment. A further annotation revealed that these TFs included critical members involved in regulating developmental processes or determining the fate of meristematic tissues. For the TFs involved in development regulation, *MYB82* is essential for trichome initiation [30]; the MADS-box TF *SVP* modulates the flowering time in response to varying temperature conditions [31]; the SBP-box TF *SPL9* plays a redundant role in regulating the transition from juvenile to adult phases [32]; and the homeobox TF *WOX13* controls the formation of replum in the Arabidopsis thaliana fruit [33].

Regarding the pivotal members that orchestrate the destiny of meristematic tissues, the myb TF *AS1* plays a crucial role in guiding leaf patterning and facilitating stem cell functions in Arabidopsis [34]; the homeobox TF *STM* is essential for the formation of the shoot apical meristem during embryonic development [35]; the auxin response TF *MP* is required to establish the embryonic axis by determining the root and vascular structures [36]; and the Homeodomain–Leucine Zipper TF *CNA* regulates stem cell definition and organ formation [37]. Additionally, these TFs potentially interact, suggesting that the loss of apical dominance observed post-HS treatment might be associated with the co-upregulation of these critical TFs that govern the fate of meristematic tissues.

### 3.3. Limitations of This Study

In conclusion, while this research provides significant insights into the impact of post-harvest HS on potato tuber sprouting, it is important to recognize the limitations of this study. Firstly, the research was primarily conducted under controlled experimental conditions, which may not fully replicate the complex natural environmental stress conditions. Future research should aim to conduct experiments under more variable and natural environmental conditions, to understand better how PHHS affects potato tubers in real-world scenarios. Secondly, this study focused on a single potato variety; thus, the responses to HS may vary among different cultivars. Subsequent research should include a broader range of cultivars. This will enable an understanding how different genetic backgrounds influence the response to HS, leading to more comprehensive and generalizable insights. Thirdly, the current analysis was limited to specific time points during the HS treatment, potentially not capturing the complete temporal dynamics of gene expression and pathway activation. Future studies should consider a more extensive temporal analysis. This could involve taking samples at more frequent intervals during and after the HS treatment to provide a more detailed view of the temporal changes in gene expression. Finally, although the critical DEGs and pathways have been identified, the direct causal relationships and underlying molecular mechanisms require further investigation. In particular, validating these genes through advanced genetic manipulation techniques, such as over-expression, silencing, or employing CRISPR-Cas9 mutagenesis in potatoes, followed by monitoring the sprouting and dominance phenotypes, will be crucial. Nonetheless, challenges, such as the potentially lethal effects caused by gene manipulation or functional redundancy, may complicate the elucidation of gene functions. Employing tissue-specific manipulations could be a strategic approach with which to circumvent these issues, thereby enabling a more precise understanding of the roles of individual genes.

## 4. Materials and Methods

### 4.1. Plant Growth and Tuber Treatments

The diploid potato breeding line AC142–01, distinguished by its short growing season, brief dormancy period, and vigorous growth, was utilized in the current research [38]. Potato plants were cultivated in a greenhouse with a 12 h light/12 h dark cycle, experiencing light intensities varying from 400 to 1000 μmol m^−2^s^−1^ within a temperature range of 18 to 25 °C. The pot size for the potatoes grown in the greenhouse was 30 cm in diameter and 35 cm in depth. The growing medium used was a mix of soil, coconut coir, and commercial organic fertilizer (powder) in a 4:2:1 ratio. Potato tubers were harvested once the plants had fully senesced. After harvest, the tubers were placed in dark conditions at 20 °C for two weeks to undergo further post-maturation before further treatment. Tubers free from mechanical damage, pests, and diseases, of a consistent size of 3–4 cm diameter, and weighing 30–50 g were selected for the HS treatment and subsequent sprouting observations. The experiment was conducted in the spring of 2022. For each treatment time point, approximately 12 tubers were used, with about 6 tubers designated for phenotypic observation and the remaining 6 for sampling the apical bud meristematic tissue. The apical bud meristematic tissue samples were collected from the central area of the eye of the apex bud, excluding the sprout, with a diameter of 4 mm and a thickness of 2 mm. Approximately two tubers’ apical bud meristematic tissues were pooled for a single sample.

The HS treatment refers to placing the tubers in dark conditions at 30 °C, with the specific treatment details illustrated in Figure 1A. Specifically, the control group underwent no HS treatment (designated as HS for 0d). The “HS for 3d” group was subjected to HS treatment for 3 days before being transitioned to standard conditions (20 °C in the dark), and so on, with the maximum treatment duration being 18 days. Photography was conducted 9 days after the treatment’s conclusion, and the sprouting length and number were documented. Photographs were taken using a Nikon D5600 DSLR camera equipped with a macro lens to capture high-resolution close-up images. The images were captured at a resolution of 24 megapixels, with a focal length of 50 mm and an aperture setting of f/8 to ensure a consistent depth of field. Measurements were conducted using ImageJ software (https://imagej.net/ij/, accessed on 22 January 2024), where the lengths of the sprouts were calibrated against a known scale, and the number of sprouts was manually recorded. Regarding the documenting of sprouting, we quantified sprouts with a length exceeding 1 mm.

Additionally, a separate batch of samples were designated for apical bud meristematic tissue sampling, with sampling every three days, starting at day 0, during the 0–18 days of HS treatment. The tubers used in the sampling were discarded after the process. Tissue samples were immediately frozen in liquid nitrogen post-collection and stored at −80 °C for future use.

### 4.2. RNA Extraction and Quantitative Real-Time PCR

RNA from potato apical bud meristematic tissue was extracted utilizing a Plant Total RNA Kit (ZOMANBIO, ZP405-1, Beijing, China), adhering to the guidelines provided by the manufacturer. The extracted RNA underwent electrophoresis on a 1% agarose gel to evaluate RNA degradation and potential contamination. The RNA concentration was determined using the Nanodrop System Nanophotometer (Implen, Munich, Germany), while RNA integrity was assessed utilizing the Agilent Bioanalyzer 2100 System (Agilent Technologies, Santa Clara, CA, USA). The synthesis of the first-strand cDNAs was facilitated using the 5× All-In-One RT MasterMix (ABM, Inc., Richmond, BC, Canada). The gene-specific primers for the potato *dUTPase* were as follows: the forward primer (5′ to 3′ orientation) was TCCCTGAAATCCCCTTTTTC, and the reverse primer (5′ to 3′ orientation) was AGGAACAGCGATGCTGAGAT. The internal reference for qRT-PCR was the potato *Ef1α* (XM_006343394; Soltu.DM.06G005580.1). Primers for validating the RNA-seq data with qRT-PCR measurements are listed in Table S2. The qRT-PCR assessments were undertaken using the LightCycler 480 system (Roche Applied Science, Mannheim, Germany) and EvaGreen Express 2× qPCR Master Mix (ABM, Inc., Richmond, BC, Canada). The computation of relative expression levels was performed as delineated in previous studies [39]. Briefly, gene expression levels were quantified utilizing the 2^–ΔΔCq^ method for the analysis.

### 4.3. Library Construction and RNA-Seq

Approximately 2 μg of RNA was utilized for library preparation. Library construction and mRNA sequencing were conducted by Wuhan Benagen Tech Solutions Limited, based in Wuhan, China. RNA sequencing procedures were executed utilizing the DNBSEQTM system from BGI in Shenzhen, China. A total of 21 libraries were analyzed, encompassing three biological replicates at each time point.

### 4.4. DEGs Analysis

Clean reads were acquired using the methodology outlined in a previous study [40]. Briefly, clean reads were acquired by removing adapter sequences, filtering out reads containing poly-N, and excluding low-quality reads. The updated DM1-3 v6.1 transcript reference (http://spuddb.uga.edu/dm_v6_1_download.shtml, accessed on 22 January 2024) was utilized, and transcript quantification was carried out utilizing Salmon, adhering to the default settings [41]. The normalization and analysis of the DEGs were facilitated through the DESeq2 package available in R [42]. DEGs of significance were pinpointed by establishing a threshold comprising an FDR <0.01 and an absolute log2 (fold change) value exceeding 1. Transcripts Per Million (TPM) effectively mitigated the impact of variations in gene lengths and sequencing disparities during the assessment of gene expression, facilitating a more accurate comparison of gene expression across different samples. The PCA analysis was conducted based on the TPM values of the top 5000 genes. The ComplexUpset package was employed to analyze the intersecting number of DEGs across various HS treatment durations.

### 4.5. KEGG and GO Enrichment Analysis

Based on a hypergeometric test, KEGG pathway enrichment analysis was performed using Tbtools [43], with an adjusted *p*-value cutoff of < 0.05. GO enrichment analyses were implemented utilizing the BiNGO plugin available in CYTOSCAPE (https://cytoscape.org, accessed on 22 January 2024) [44], maintaining the threshold defined by the Benjamini and Hochberg FDR of below 0.01. The GO gene sets that cleared rigorous significance thresholds (FDR < 0.01) were subjected to an in-depth analysis with the assistance of the EnrichmentMap plugin tool within CYTOSCAPE, aiding in the generation of the enrichment map [45].

### 4.6. Cluster and Heatmap Analysis

For the clustering process, the mean value of the TPM for each treatment was standardized to have a zero mean value and a standard deviation of one for each gene’s profile. The transformed expression data were then segmented using the fuzzy c-means (FCM) clustering method, incorporated into the Mfuzz package [46]. This method assigns a membership value between 0 and 1 to each gene as a marker of how indicative a gene profile is for a specific cluster. The clustering method employs Euclidean distance as a similarity measurement criterion. It necessitates two primary parameters: ‘c’, which indicates the number of clusters, and ‘m’, a parameter in the FCM that governs the extent of noise influence on the cluster analysis. After iterative refinement, the optimum values assigned for ‘c’ and ‘m’ in the current investigation were established as 6 and 1.75, respectively. Heatmap analysis and visualization were performed using the ComplexHeatmap package [47].

### 4.7. Functional Network Analysis

Network clusters were discerned utilizing the STRING website (https://string-db.org, accessed on 22 January 2024), applying the potential core regulatory transcription factors and their associated proteins identified in Cluster 6.

### 4.8. Statistical Analysis

Statistical analysis was conducted using GraphPad Prism 10 software, employing one-way ANOVA followed by Tukey’s multiple comparisons test.

## 5. Conclusions

In conclusion, this study elucidates that post-harvest HS promotes potato tuber sprouting and apical dominance release. The transcriptomic scrutiny highlighted potential regulatory pathways orchestrated through variations in the gene expression patterns concerning starch metabolism, hormonal biosynthesis, chromosomal organization, and developmental modulation. The present study’s delineation of the critical genes and pathways augments comprehension of the complex interplay between HS and meristem functionality, thereby enhancing the understanding of the acceleration of sprouting processes in potato tubers. These findings also provide a reference for understanding the mechanism of premature sprouting caused by pre-harvest HS. This study likely represents a pioneering assessment of the impact of post-harvest HS treatments on potato sprouting, tracking the transition from dormancy to sprouting through a time-series sampling approach. Further research, focusing on the genetic transformations of the identified crucial genes that may regulate potato sprouting, could significantly advance our understanding of the regulatory mechanisms. Such insights are crucial for developing strategies to control post-harvest sprouting, enhancing potato quality and shelf-life management. For instance, applying gene editing strategies to manipulate the critical genes involved in sprouting control could effectively inhibit post-harvest sprouting in potatoes. This approach deepens the comprehension of potato biology and holds practical applications for the agricultural sector.

## Data Availability

All relevant data can be found within the manuscript and its supporting materials.

## Supplementary Materials

The following supporting information can be downloaded at: https://www.mdpi.com/article/10.3390/ijms25031699/s1.
