# Supplementary material for: Molecular Insights into the Accelerated Sprouting of and Apical Dominance Release in Potato Tubers Subjected to Post-Harvest Heat Stress
Source: Int J Mol Sci. 2024 Jan 30;25(3):1699. doi: 10.3390/ijms25031699 (PMC10855572; doi:10.3390/ijms25031699)
Supplement: Supplementary file 1 [file ijms-25-01699-s001.zip › Supplementary Figures.pdf]

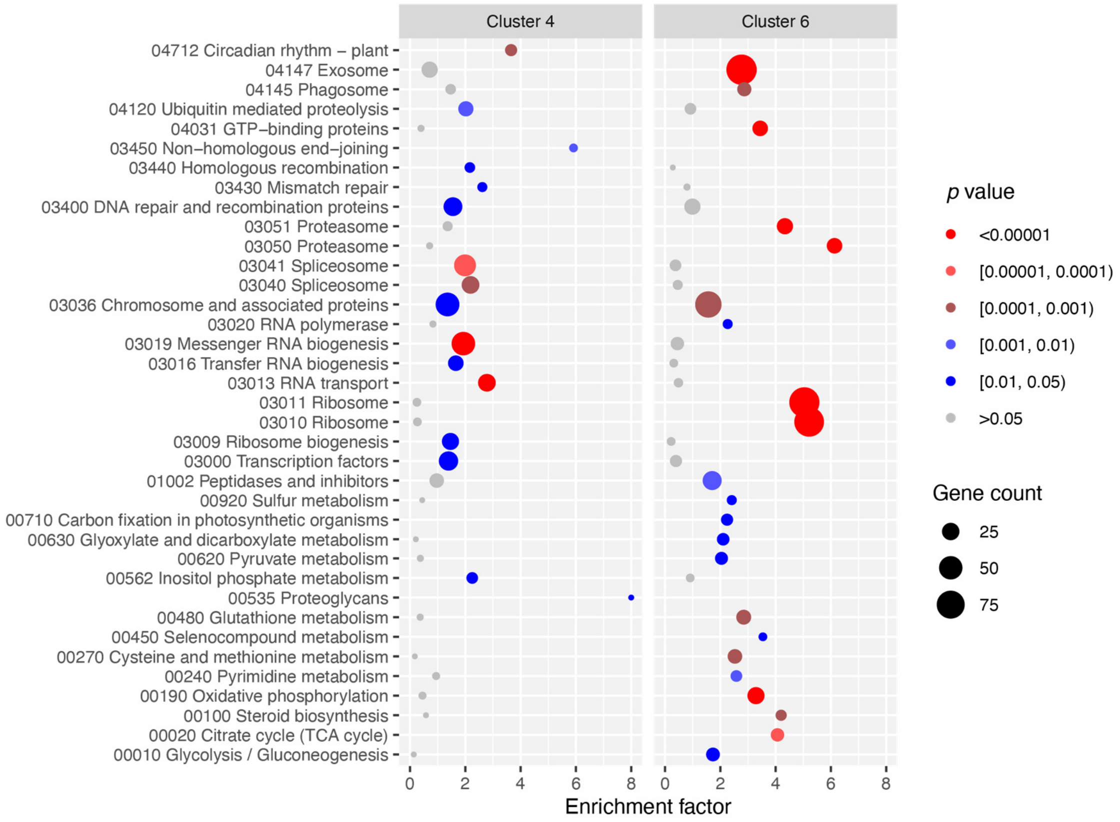

Figure S1. KEGG enrichment analysis of DEGs in Cluster 4 and 6.

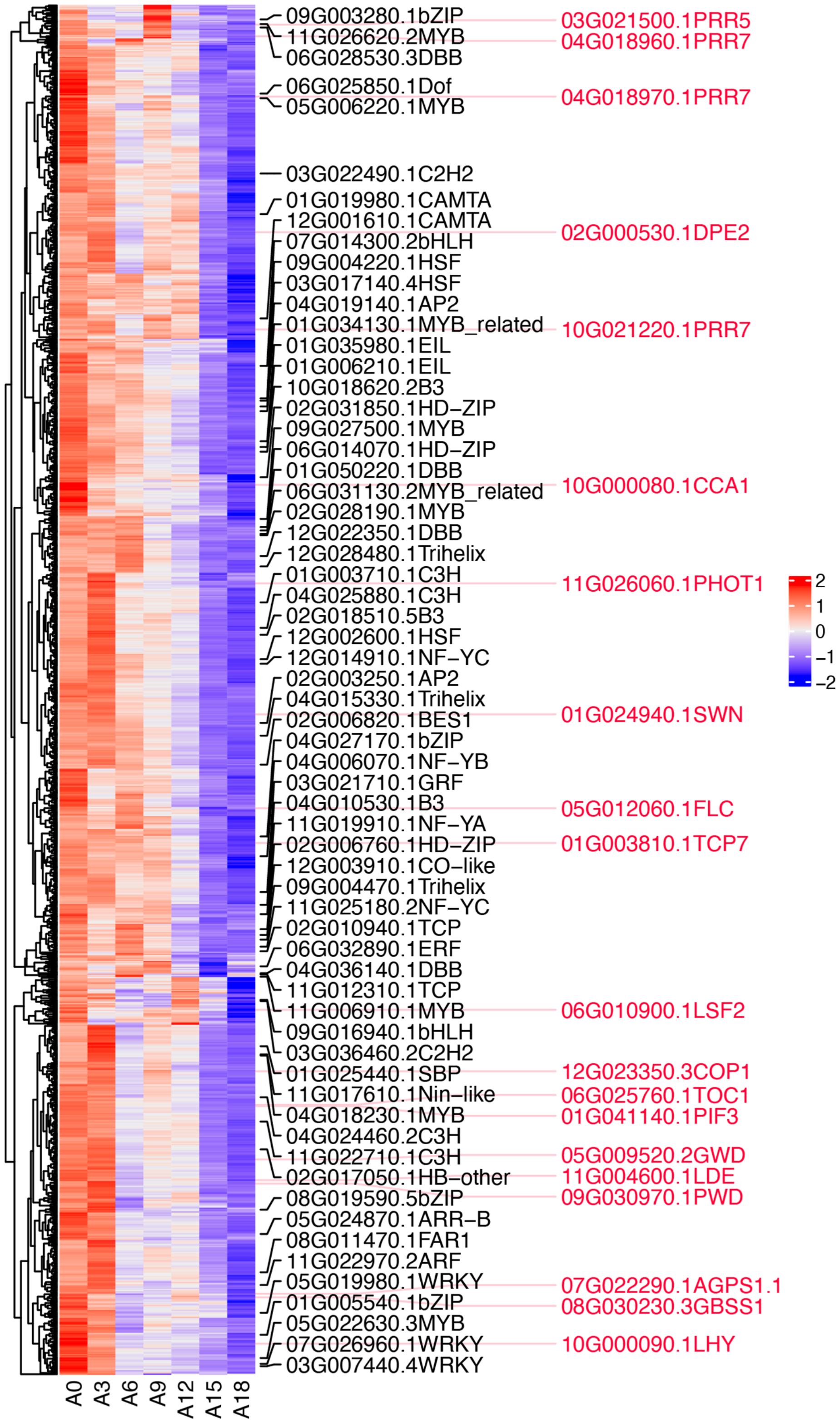

Figure S2. Heatmap illustration of DEGs in Cluster 4. DEGs encoding TFs, histones, and genes implicated in the "Starch Metabolic Process", "Photomorphogenesis", and the "Circadian Rhythm-Plant" are highlighted.

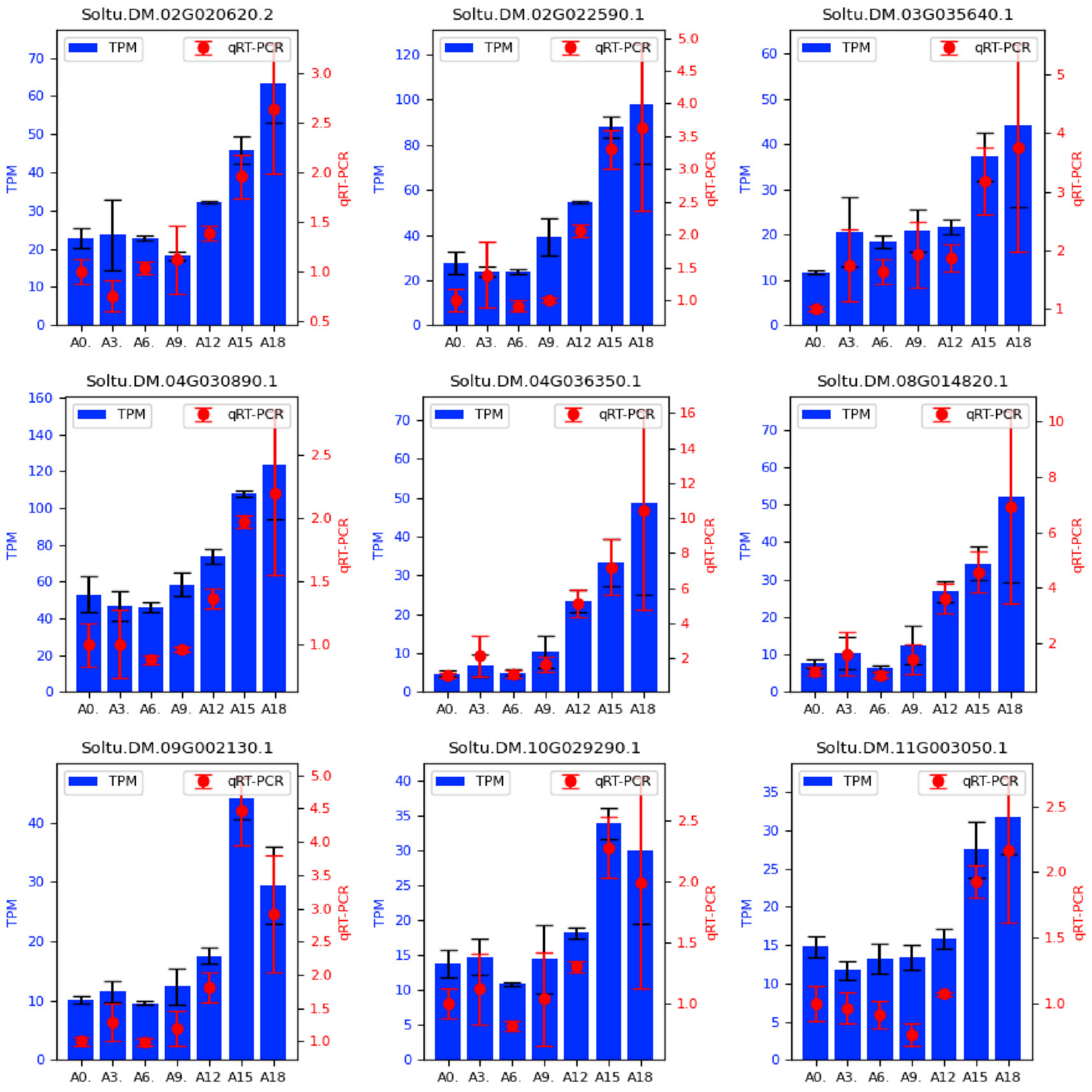

Figure S3. Validating the RNA-seq data with qRT-PCR measurements. The blue bars demonstrate the expression levels quantified by TPM (Transcripts Per Million), while the red points with error bars represent the expression levels as quantified by qRT-PCR. Data are presented as means ± SD (n=3).

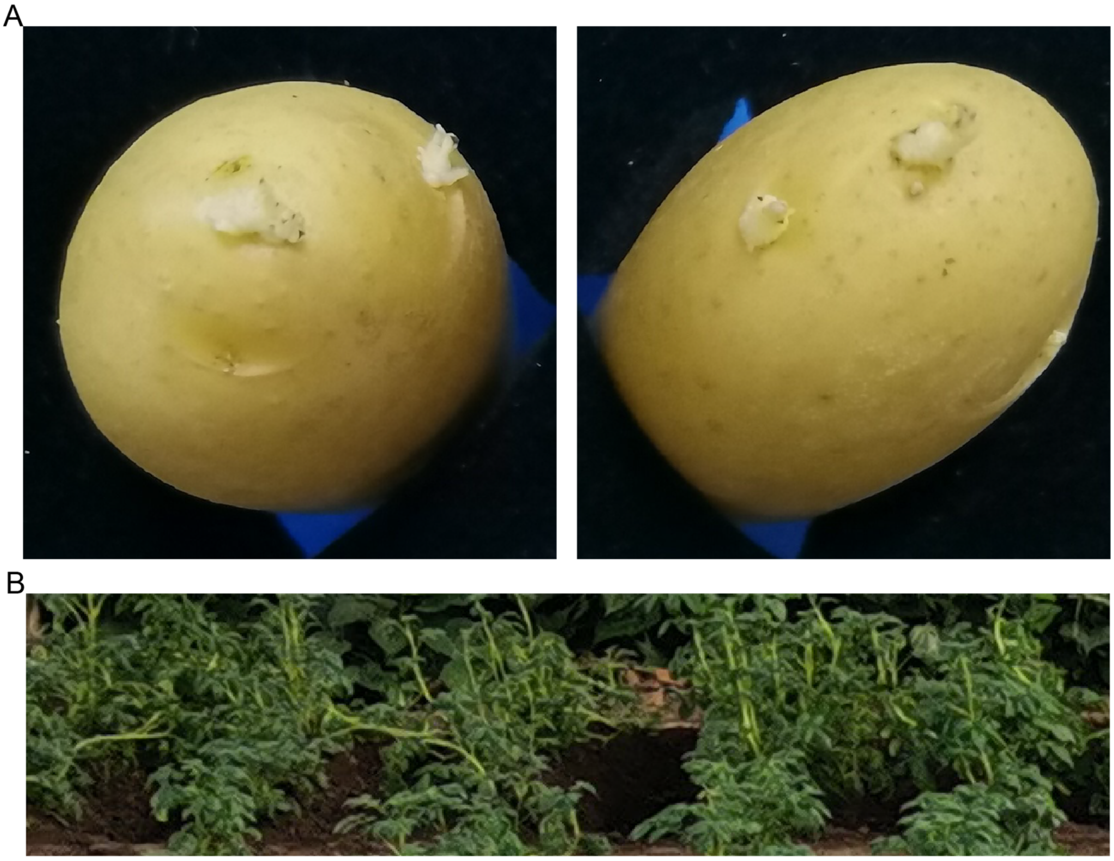

Figure S4. The effects of post-harvest HS treatment on subsequent sprouting growth and plant growth. (A) Following the cessation of HS treatment after 18 days, the growth status of sprouting was assessed at 16 days post-treatment. (B) Utilizing seed potatoes that underwent HS treatment, the growth conditions in the field were documented (photographs taken approximately 30 days post-emergence).
